# Rotational varifocal moiré metalens made of single-crystal silicon meta-atoms for visible wavelengths

**DOI:** 10.1515/nanoph-2021-0690

**Published:** 2022-01-14

**Authors:** Chikara Ogawa, Sotaro Nakamura, Takumi Aso, Satoshi Ikezawa, Kentaro Iwami

**Affiliations:** Department of Industrial Technology and Innovation, Tokyo University of Agriculture and Technology, Koganei, Tokyo 184-8588, Japan; Department of Mechanical System Engineering, Tokyo University of Agriculture and Technology, Koganei, Tokyo 184-8588, Japan

**Keywords:** dielectric metasurface, metalens, silicon, varifocal, waveguide

## Abstract

Metasurface lenses (metalenses) offer an ultrathin and simple optical system with dynamic functions that include focal length tuning. In this study, a rotational varifocal (i.e., moiré) metalens based on octagonal single-crystal silicon pillars was designed and fabricated to realize a high transmittance, whole 2*π* phase coverage, and polarization insensitivity for visible wavelengths. The moiré metalens consists of a pair of cascaded metasurface-based phase lattices and the focal length can be adjusted from negative to positive by mutual rotation. The fabricated moiré metalens demonstrated a focal length that can be tuned from −36 mm to −2 mm and from 2 to 12 mm by mutual rotation from −90° to 90°, and the experimental measurements agreed well with theoretical values at the design wavelength of 633 nm. Imaging was demonstrated at three distinct wavelengths of 633, 532, and 440 nm.

## 1 Introduction

Metasurfaces are a planar type of metamaterial and are promising as optical components. They can be used to tailor a transmitted wavefront, and they have attracted attention because of their easy fabrication and wide applicability to various devices [1–3]. Metasurfaces can be applied in retarders and waveplates [4–8], vector beam converters [9–11], color filters [12–14], and holography [15–19].

Metasurface lenses, which are also known as metalenses, have attracted considerable attention for their ultrathin and lightweight characteristics and their broad applicability [20–27]. Metalenses have been used to pioneer novel optical functions for lenses, including polarization imaging and polarimetry [28–31], wavelength routing [32], spectrometers [33], and ranging [34]. A tunable focal length (i.e., varifocal) is one of the most important characteristics required for lens systems. Various mechanisms for varifocal metalenses have been studied, such as microelectromechanical system (MEMS)-actuated longitudinal motion [33], expansion of a polydimethylsiloxane matrix [35], temperature-sensitive polymers [36], and lateral motion (i.e., Alvarez metalens) [37–40].

Rotational varifocal metalenses, which are known as moiré metalenses, consist of a pair of two metasurfaces. Their focal length can be tuned by mutual rotation. Moiré metalenses have the advantages of compactness along the optical axis, an unchanged aperture size, and a wide tuning range from negative to positive focal lengths. Following the development of diffractive optical elements (DOEs) [41–45], moiré metalenses have been demonstrated at microwave [46], infrared-B [47], and infrared-A [48] wavelengths. Luo et al. [49] recently demonstrated a moiré metalens for the visible wavelength of 532 nm, although its tuning range was limited to the positive region.

In this study, a moiré metalens for visible wavelengths and with both negative and positive focal length tuning was designed and fabricated by using single-crystal silicon octagonal pillars. At the designed wavelength of 633 nm, adjusting the mutual rotation angle by ±90° resulted in a tuning range from −36 mm to −2 mm and from 2 to 12 mm. Imaging at three distinct wavelengths of 633, 532, and 440 nm was demonstrated.

## 2 Theory and design

The phase profile *ϕ*(*r*) of a convex spherical lens can be expressed by paraxial approximation:

(1) $$\phi \left(r\right)=\frac{2\pi }{\lambda }\left(\sqrt{f^{2}+r^{2}}-f\right)\approx \frac{\pi r^{2}}{\lambda f},$$

where *r* is the radial coordinate, *λ* is the wavelength, and *f* is the focal length.

**Figure 1:**
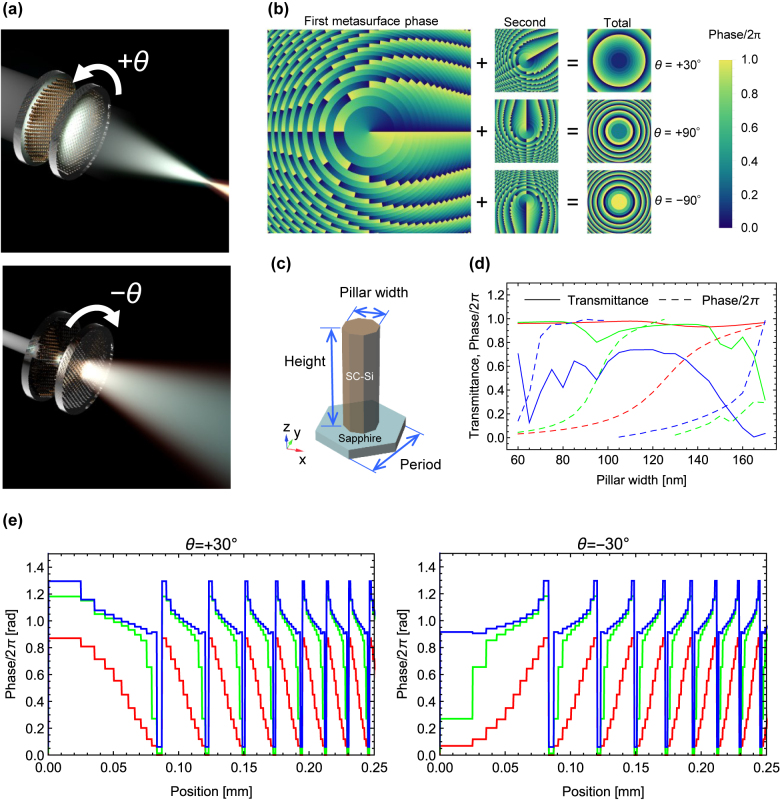
Design principle of a rotational varifocal (i.e., moiré) metalens. (a) Schematic drawing of focal length tuning. (b) Phase profiles of each metasurface and the overall metalens at different mutual rotation angles. (c) Schematic of an octagonal pillar meta-atom made of single-crystal in a hexagonal arrangement. (d) Simulated transmission property as a function of the pillar width. Solid and dashed lines indicate the transmittance and phase/2*π*, respectively. The plot colors of red, green, and blue correspond to the wavelengths of 633, 532, and 442 nm, respectively. (e) Phase profiles around the center of the designed moiré metalens with the diameter of 2 mm at mutual rotation angles of +30° (left) and −30° (right) and wavelengths of 633 (red), 532 (green), and 440 (blue) nm.

Figure 1 shows the design principle of a moiré metalens. As shown in Figure 1a, the moiré metalens consists of a pair of two superimposed metasurfaces, and their mutual rotation angle can be adjusted to tune the focal length over a wide range from negative to positive values. The two metasurfaces have the phase profiles of *ϕ*_first_ and *ϕ*_second_, which can be expressed as follows in polar coordinates (*r*, *φ*) [41]:

(2) $$\begin{array}{cc} \phi_{\text{first}}\left(r,\varphi \right) & =round\left(ar^{2}\varphi \right)\\ \phi_{\text{second}}\left(r,\varphi \right) & =-round\left(ar^{2}\varphi \right), \end{array}$$

where *a* is a constant. Equation (2) shows that the second metasurface has the phase profile of the first one but upside down. With the mutual rotation angle *θ*, the phase profile of the moiré metalens *ϕ*_moiré_(*θ*) is given by:

(3) $$\begin{array}{cc} \phi_{\text{moire}} & =\phi_{\text{first}}\left(r,\varphi \right)+\phi_{\text{second}}\left(r,\varphi -\theta \right)\\ & =round\left(ar^{2}\theta \right). \end{array}$$

This means the total phase profile of the moiré metalens is similar to that of a spherical lens. Note that the round operator is applied to avoid the sectoring effect [41]. If Eqs. (1) and (3) are compared, they show that the focal length *f*(*θ*) of the moiré metalens can be tuned by changing *θ*:

(4) $$f^{-1}\left(\theta \right)=\frac{a\lambda \theta }{\pi }.$$

Figure 1b shows the phase profile of the moiré metalens at different rotation angles of +30°, +90° and −90°. The former two are convex lenses with different focal powers, and the latter is a concave lens.

In Eq. (4), *θ* has a range of ±180°. However, the efficiency of the moiré metalens decreases at large rotation angles because of the bifocal effect. Therefore, to obtain a diffraction efficiency of >85% when using transparent meta-atoms, the range of *θ* should be limited to within ±90° [41]. Under this condition, the achievable maximum numerical aperture (NA) is expressed by

(5) $${\text{NA}}_{\max}=\frac{2p}{\lambda },$$

where *p* is the period of meta-atom expressing the phase profile.

Similar to diffractive lenses, moiré metalens have a negative chromatic aberration. According to Eq. (4), the moiré metalens can be designed to have a reference focal length *f*_0_ at a certain wavelength *λ*_0_. Then, the focal length *f* at the wavelength *λ* is expressed by

(6) $$f\left(\lambda \right)=\frac{f_{0}\lambda_{0}}{\lambda }.$$

The spacing between the two lenses is an important factor. When the spacing is large, the phase profile created by the first lens changes its shape as it propagates. Before it reaches the second lens, the shape differs from the ideal as given in Eq. (2), which reduces the focusing efficiency and image quality. To optimize the performance of the moiré metalens, the spacing should be kept smaller than the Talbot length *L*_Talbot_ = 2*p* ^2^/*λ* [41]. However, this is challenging to achieve in experiments. Qian et al. [50] recently reported a reinforced design method for moiré metalens that allows for a large separation.

The material selection of the meta-atoms is also a critical issue for dielectric metalenses. Materials with a wide bandgap are often used for waveguide-type meta-atoms working in visible wavelengths, including titanium oxide [24, 26, 27], silicon nitride [31, 39], and gallium nitride [32]. However, an increasing bandgap tends to correspond to a decreasing refractive index, which requires increasing the aspect ratio and applying the Pancharatnam–Berry phase to achieve 2*π* phase coverage at the expense of efficiency. On the other hand, because silicon is an indirect semiconductor, its extinction coefficient does not become very high even at wavelengths greater than the bandgap, especially in single crystals (*N* = 3.8774 + 0.00099*i* at 633 nm [51]).

In this study, octagonal pillars made of single-crystal silicon (SC-Si) were used as meta-atoms, as shown in Figure 1c. An octagonal shape was adopted to apply the character-projection (CP) method to electron-beam (EB) lithography for high-throughput fabrication. Pillars with a height of 300 nm were arranged in a hexagonal lattice with a period of 260 nm. Figure 1d shows the phase shift and transmittance as a function of the pillar width ranging from 80 to 180 nm at wavelengths of 633, 532, and 442 nm, which was calculated by using commercially available finite element software (COMSOL 5.1, COMSOL Inc., USA). A full 2*π* phase shift can be achieved at all three wavelengths, especially with high transmittance at 633 and 532 nm. These pillars were utilized to design a moiré metalens with a diameter of 2 mm. The parameter *a* in Eq. (4) was set to 1.580728 × 10^−9^ rad^−1^ nm^−2^ to achieve a focal length of 2 mm (NA = 0.5) at the mutual rotation angle of +90° and the design wavelength of 633 nm.

Figure 1e shows the phase profile around the center of the designed moiré metalens with the diameter of 2 mm at mutual rotation angles of +30° (left) and −30° (right) and wavelengths of 633 (red), 532 (green), and 440 (blue) nm. A smaller wavelength resulted in a smaller phase gradient and thus a longer focal length.

**Figure 2:**
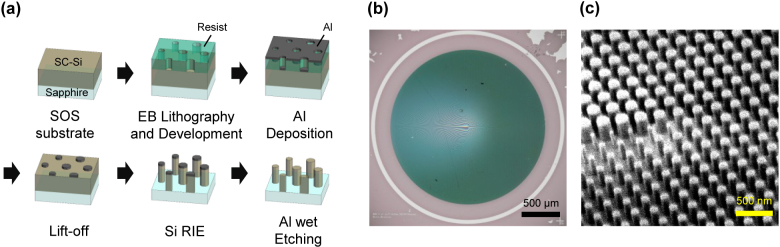
Fabrication of the moiré metalens. (a) Fabrication process flow. (b) Microscope image of the fabricated metasurface with a diameter of 2 mm on the sapphire substrate. (c) SEM image of the center of the fabricated metasurface.

Figure 2a shows the fabrication process of the designed moiré metalens. A commercially available silicon-on-sapphire (SOS) wafer was used, where a single-crystal silicon (100) film was epitaxially grown with a thickness of 300 nm on the R-plane of a double-side polished sapphire substrate with a thickness of 460 μm. The wafer was diced into 2 × 2 cm^2^ square chips along with the orientation flat (45° from the *c*-axis projected on the R-plane). Then, the lens pattern was drawn by direct-writing EB lithography (F7000S-VD02, Advantest, Japan). Layout data files were prepared using a Python library gdstk. The ZEP 520A-7(ZEON Co., Japan) resist was used with a surfactant (hexamethyldisilazane; HMDS) and antistatic agent (ESPACER 300Z, Showa Denko Co., Japan). Most of the meta-atom patterns were drawn by using the CP method, which uses dedicated stencil masks for e-beam shaping. This method drastically reduces the number of shots and achieves a high drawing throughput. Thus, a 2-mm-diameter moiré metalens pattern consisting of more than 30 million meta-atoms with a nominal width across flats ranging from 60 to 170 nm in 10 nm increments was written in less than 10 min. After the development of the resist, the pattern was transferred to a vacuum-evaporated aluminum film through the lift-off process. Then, the aluminum patterns were used as an etching mask to form the silicon pillars by inductively-coupled plasma reactive ion etching (RIE) apparatus. Finally, the aluminum mask was removed by wet etching.

Two identical metasurfaces were fabricated, and they were superposed with one flipped upside down to make the moiré metalens. A photograph of the fabricated metasurfaces for the moiré metalens is shown in Figure 2b. Figure 2c shows the scanning electron microscope (SEM) image of the pillars around the center of the metalens. Width-distributed pillars with vertical sidewalls were successfully fabricated.

## 3 Results and discussions

**Figure 3:**
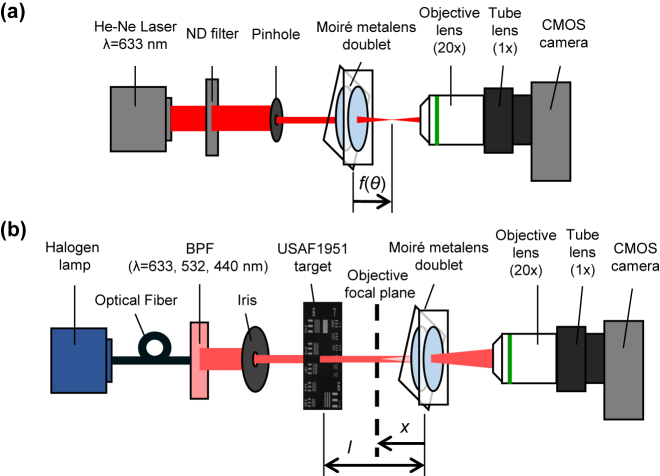
Experimental setup for the performance evaluation of metalens: (a) for positive mutual rotation angles and (b) for negative mutual rotation angles.

Figure 3 shows the experimental setups for the focal length measurement and imaging characterization. The focusing behavior at the design wavelength of 633 nm was evaluated by obtaining focal spot images with the setup shown in Figure 3a. A He–Ne laser (*λ* = 633 nm) was used with a neutral density filter and the pinhole. One of the metasurfaces of the moiré metalens was mounted on a manual rotation mount (CRM1T, Thorlabs Inc., USA), which could change the mutual rotation while maintaining the in-plane alignment between two layers. The focal spot images were captured by using a monochrome complementary metal-oxide-semiconductor (CMOS) camera (DCC1545M, Thorlabs Inc., USA) with a 20× objective lens (M-PLAN APO 20× NA = 0.42, Mitutoyo, Japan) and a 1× tube lens (MT-40, Mitutoyo, Japan). Because the moiré metalens also works as a concave lens, negative focal lengths were determined by observing images of a resolution test target using the setup shown in Figure 3b at three distinct wavelengths: 633 nm (i.e., the design wavelength), 532 nm, and 440 nm. A tungsten halogen white light source was used to avoid laser speckle noise. Then, the incident light passed through one of three bandpass filters for wavelengths of 633 nm (FL632.8-3, Thorlabs Inc., center wavelength = 632.8 nm, FWHM = 3 nm), 532 nm (FL532-3, Thorlabs Inc., center wavelength = 532 nm, FWHM = 3 nm), and 633 nm (FB440-10, Thorlabs Inc., center wavelength = 440 nm, FWHM = 10 nm), as well as the resolution test target (USAF 1951). The virtual images of the USAF target were obtained by using the same CMOS camera and the 20× objective lens. The CMOS camera was mounted on the micrometer stage, and its position *x* was measured in steps of 0.05 mm. Using the distance *l* between the metalens and the test target and the image plane position *x* from the metalens shown in Figure 3b, the focal length *f*(*θ*) of the moiré metalens at the mutual rotation angle *θ* was calculated as follows:

(7) $$\frac{1}{f\left(\theta \right)}=\frac{1}{l}+\frac{1}{x}$$

where *l* was set to 27.16 mm.

**Figure 4:**
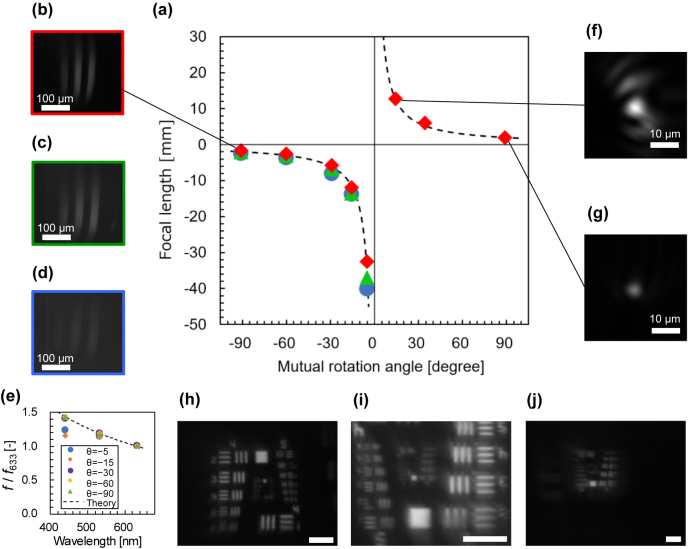
Measurement results. (a) Measured focal length as a function of the mutual rotation angle and (b–i) imaging results. (b–d) show images captured at the same mutual rotation angle of −90.9° at wavelengths of 633, 532, and 440 nm, respectively. (e) Change in the focal length normalized to *f*_633_ as a function of the wavelength. The dashed curve shows the theoretical relationship given by Eq. (6). (f) and (g) show focal spot images captured at the mutual rotation angles of +14.4° and +88.9°, respectively. (h–j) show the USAF target images observed at a wavelength of 633 nm and mutual rotation angles of −32.0°, +28.3°, and +88.5°, respectively. The scale bars in (h–j) indicate a distance of 300 μm.

Figure 4a summarizes focal length measurement results. The red rhombuses, green triangles, and blue circles correspond to the results at the wavelengths of 633, 532, and 440 nm, respectively. The dashed curve indicates the design characteristics of *f*_633_(*θ*) = *π*/(*aλθ*) at 633 nm. The results and design agreed well for both the negative and positive focal length ranges. For the negative focal lengths, at the same mutual rotation angle, a shorter wavelength resulted in a larger image and greater focal length. For example, Figure 4b–d show photographs of the USAF target at a rotation angle of −90.9° and wavelengths of 633, 532, and 440 nm, respectively. Figure 4e plots the change in focal length (normalized to *f*_633_) versus wavelength for each rotation angle. The measured results showed good agreement with the theoretical curve, which was inversely proportional to the wavelength as shown in Eq. (6).

Figure 4f and g show the focal spot of the He–Ne laser beam at the rotation angles of +14.4° (f) and +88.9° (g), respectively. Pinholes with diameters of 2 and 0.3 mm were used to obtain Figure 4f and g, respectively. Figure 4f shows orientation-dependent diffraction patterns around the focal spot. This was attributed to the distortion caused by the misalignment between the two metasurfaces. The misalignment was measured to be 2 μm (not shown). As reported in previous papers [41, 49], a misalignment between two components of a moiré lens does not affect to the focal length, whereas decreases the image quality and focusing efficiency. Figure 4h–j show captured images at a wavelength of 633 nm and rotation angles of −32.0°, +28.3°, and +88.5°, respectively. Figure 4h shows a straight virtual image, which corresponds to a negative focal length. Meanwhile, Figure 4i and j show inverted real images, which correspond to positive focal lengths. Although these images are slightly blurred, the patterns of groups 4 and 5 on the USAF target are clearly visible. A possible reason for the blurring is the distance between the two metasurfaces of moiré metalens. In this study, the distance was 60–140 μm because a Kapton tape (50 μm thickness) was used for adhesion. Simulations of moiré metalenses, such as those by Qian et al. [50], have shown that a large spacing may cause a subfocal spot to form beside the focal point, which can blur the image. Note that the effect of birefringence of the sapphire substrate was negligible. This was confirmed by observation when a polarizer was inserted.

The diffraction efficiency of the metalens was also evaluated. At a rotation angle of *θ* = +15°, the power of the transmitted light was measured by using a power meter (PM100USB + S120C, Thorlabs Inc.,) at the focal plane (*P*_f_: 12 mm away from the lens) and at the far field (*P*_far_: 200 mm away). The measured diffraction efficiencies ((*P*_f_ − *P*_far_)/*P*_f_) were 64, 25, and 8% at the wavelengths of 633, 532, 440 nm, respectively. The decrease in diffraction efficiency at 633 nm compared to the theoretical value (>85%) can be attributed to the increased distance between the two lens components.

In conclusion, the designed moiré metalens can be tuned from negative to positive focal lengths at visible wavelengths. The moiré metalens was designed by using polarization-insensitive single-crystal silicon pillars. The tuning range of the focal length was from −36 mm to −2 mm and from 2 to 12 mm for a mutual rotation angle within ±90° at the design wavelength of 633 nm. Imaging at three distinct wavelengths of 633, 532, 440 nm was also demonstrated. The improvement of chromatic aberration should be the next issue, which is expected to be solved by following the strategy used for diffractive lenses [44, 45].
